# Trim25 Is an RNA-Specific Activator of Lin28a/TuT4-Mediated Uridylation

**DOI:** 10.1016/j.celrep.2014.10.017

**Published:** 2014-11-06

**Authors:** Nila Roy Choudhury, Jakub S. Nowak, Juan Zuo, Juri Rappsilber, Steven H. Spoel, Gracjan Michlewski

**Affiliations:** 1 Wellcome Trust Centre for Cell Biology, University of Edinburgh, Michael Swann Building, Edinburgh EH9 3BF, UK; 2 Department of Biotechnology, Technische Universität Berlin, 13353 Berlin, Germany; 3 Institute of Molecular Plant Sciences, University of Edinburgh, Daniel Rutherford Building, Edinburgh EH9 3BF, UK

## Abstract

RNA binding proteins have thousands of cellular RNA targets and often exhibit opposite or passive molecular functions. Lin28a is a conserved RNA binding protein involved in pluripotency and tumorigenesis that was previously shown to trigger TuT4-mediated pre-let-7 uridylation, inhibiting its processing and targeting it for degradation. Surprisingly, despite binding to other pre-microRNAs (pre-miRNAs), only pre-let-7 is efficiently uridylated by TuT4. Thus, we hypothesized the existence of substrate-specific cofactors that stimulate Lin28a-mediated pre-let-7 uridylation or restrict its functionality on non-let-7 pre-miRNAs. Through RNA pull-downs coupled with quantitative mass spectrometry, we identified the E3 ligase Trim25 as an RNA-specific cofactor for Lin28a/TuT4-mediated uridylation. We show that Trim25 binds to the conserved terminal loop (CTL) of pre-let-7 and activates TuT4, allowing for more efficient Lin28a-mediated uridylation. These findings reveal that protein-modifying enzymes, only recently shown to bind RNA, can guide the function of canonical ribonucleoprotein (RNP) complexes in *cis*, thereby providing an additional level of specificity.

## Highlights

• Lin28a binding to a pre-miRNA is insufficient to trigger TuT4-mediated uridylation
• The E3 ligase Trim25 binds to the conserved terminal loop of pre-let-7
• Trim25 is an RNA-specific cofactor for Lin28a/TuT4-mediated uridylation

## Graphical Abstract

**Figure:**
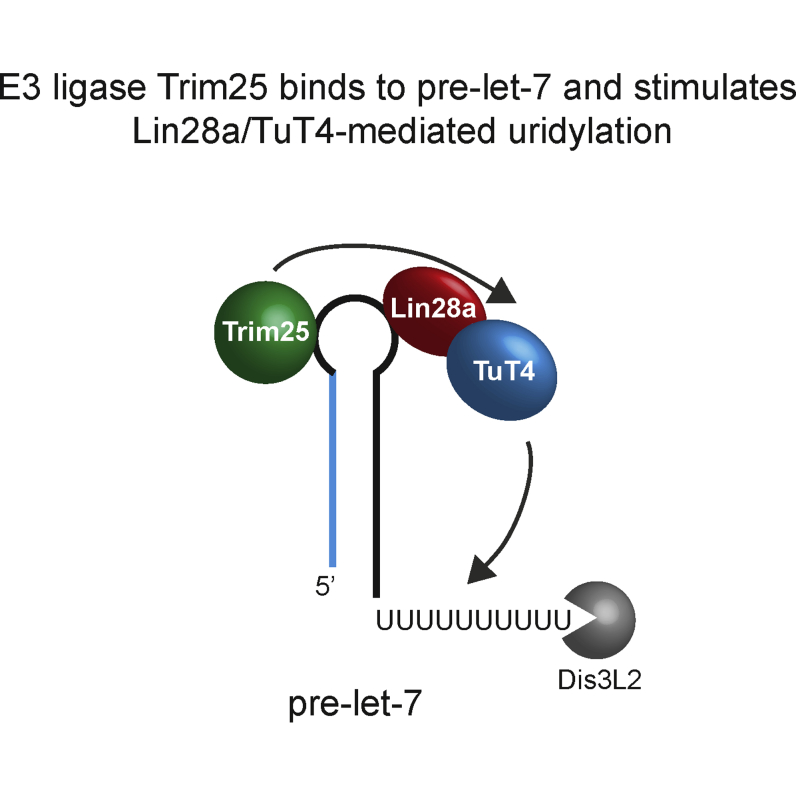

## Teaser

Lin28a triggers TuT4-mediated pre-let-7 uridylation. Despite binding to other pre-microRNAs, only pre-let-7 is efficiently uridylated by TuT4. Choudhury et al. show that Trim25 is an RNA-specific cofactor for Lin28a/TuT4-mediated uridylation. These findings reveal that Trim25 can guide the function of canonical RNP complexes in *cis*, thereby providing an additional level of specificity.

## Introduction

Next-generation studies on RNA binding proteins have supplied a constant stream of novel RNA-protein interactions (Ascano et al., 2012; Milek et al., 2012; Ray et al., 2013). Additionally, new clades of RNA binding proteins, such as kinases, E3 ligases, and metabolic enzymes, have recently been discovered by high-throughput proteomic analysis, and their RNA-related roles are awaiting characterization (Baltz et al., 2012; Castello et al., 2012; Hentze and Preiss, 2010; Kwon et al., 2013). In the light of the often short and abundant RNA sequence motifs that provide protein-binding specificity, a fundamental question is which RNA-protein complexes are functional as opposed to being promiscuous or passive.

Lin28a is an example of an RNA binding protein with a well-defined binding motif (GGAG) and roles in the control of microRNA (miRNA) maturation (Heo et al., 2009; Nowak et al., 2014; Van Wynsberghe et al., 2011; Viswanathan et al., 2008), mRNA translation (Cho et al., 2012; Shyh-Chang et al., 2013), and mRNA splicing (Wilbert et al., 2012). In undifferentiated metazoan cells, Lin28a suppresses the maturation of let-7 miRNAs by binding to the conserved terminal loop (CTL) of the let-7 precursor (pre-let-7) and recruiting the 3′ terminal uridyl transferase (TuTase) TuT4, which adds an oligouridine tail to the 3′ end of pre-let-7 (Hagan et al., 2009; Heo et al., 2009; Lehrbach et al., 2009). The oligouridine tail inhibits cleavage by Dicer (Heo et al., 2008) and targets pre-let-7 for degradation by the exonuclease Dis3L2 (Chang et al., 2013; Ustianenko et al., 2013). Other mechanisms describing the blockage of primary microRNA (pri-miRNAs) and pre-miRNA processing have also been suggested (Lightfoot et al., 2011; Piskounova et al., 2008). Strikingly, even though in vivo (Cho et al., 2012; Hafner et al., 2013; Wilbert et al., 2012) and in vitro (Towbin et al., 2013) binding data show that Lin28a binds many more pre-miRNAs, only some members from the pre-let-7 family are efficiently uridylated. Hence, we predicted the existence of substrate-specific cofactors that enable Lin28a-mediated uridylation of pre-let-7 and/or restrict its functionality on non-let-7 pre-miRNAs.

Here, we show that Lin28-mediated uridylation is dependent on the structure of the RNA and the sequence context of the Lin28a binding motif. Using RNA pull-down combined with high-throughput stable isotope labeling with amino acids in cell culture (SILAC) mass spectrometry, we discovered that the E3 ligase Trim25 is a factor that binds to pre-let-7. We found that Trim25 colocalizes and coimmunoprecipitates with Lin28a. We also found that Trim25 activates TuT4 and that drug inhibition of the E1 ubiquitin ligase reduces the levels of TuT4 and decreases pre-let-7 uridylation. Finally, using in vitro pre-miRNA processing and in vivo pre-miRNA quantification, we showed that Trim25 is an RNA-specific cofactor for Lin28a-mediated uridylation of pre-let-7. Altogether, our results uncover an important layer of RNA binding factors that can modify canonical ribonucleoprotein (RNP) complexes and control their molecular function.

## Results

### Lin28-Mediated Uridylation Is Dependent on RNA Structure and the Sequence Context of the GGAG Motif

**Figure 1:**
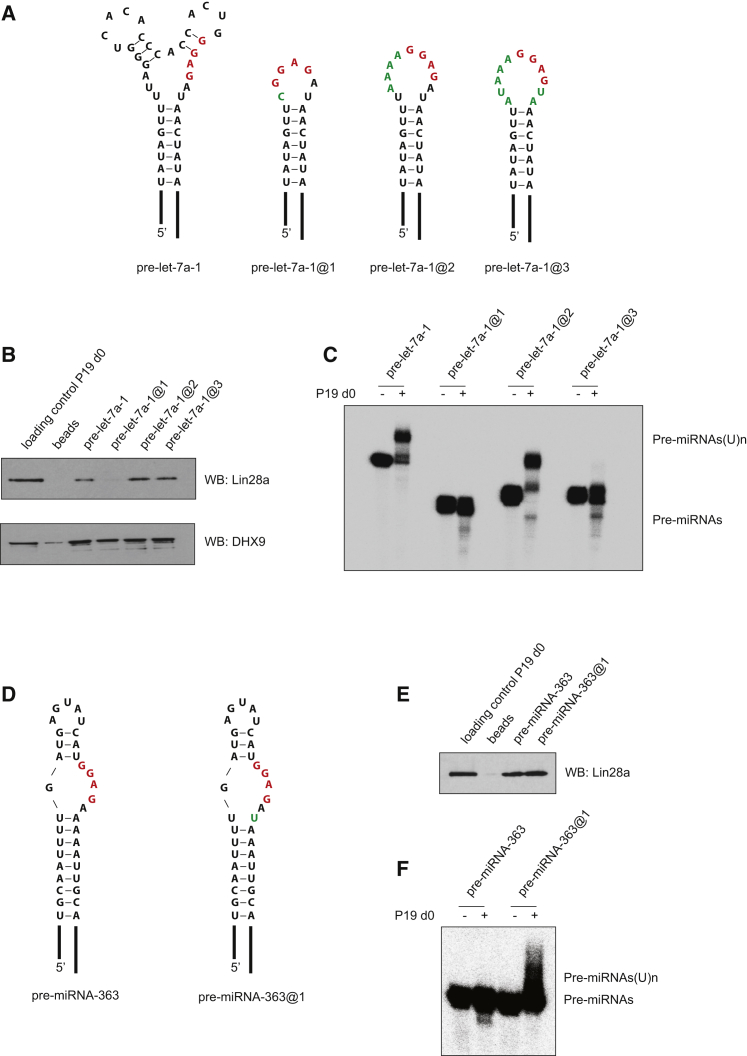
Structural and Sequence Context of the GGAG Motif Determine Lin28a Binding and Functionality (A) Schematic of the secondary structure of wild-type and conserved terminal loop (CTL) mutants of pri-let-7a-1. The mutated nucleotides are in green, and the GGAG motif is in red. (B) Western blot (WB) analysis of Lin28a and DHX9 proteins in RNA pull-downs from day 0 (d0) P19 teratocarcinoma cell extract using wild-type pre-let-7 or its CTL mutants. (C) In vitro processing uridylation assays performed with internally radiolabeled pre-let-7a transcripts (50 × 10^3^ cpm, approximately 20 pmol) in the presence of d0 P19 cell extract. (−) represents an untreated control. Reactions were supplemented with 0.25 mM UTP. The products were analyzed on an 8% denaturing polyacrylamide gel. (D) Schematic of the secondary structure of wild-type pri-miRNA-363 and its CTL mutant. (E) Western blot analysis of Lin28a protein in RNA pull-downs from day 0 P19 cell extract using wild-type pre-miRNA-363 or mutant pre-miRNA-363@1. (F) In vitro processing uridylation assays performed with internally radiolabeled pre-miRNA-363 transcripts (50 × 10^3^ cpm, approximately 20 pmol) in the presence of d0 P19 cell extracts. (−) represents an untreated control. Reactions were supplemented with 0.25 mM UTP. The products were analyzed on an 8% denaturing polyacrylamide gel.

Previously, the GGAG motif was shown to be sufficient to induce Lin28a binding, but not uridylation (Heo et al., 2009). A fixed distance between the motif and the pre-miRNA stem was shown to determine the functionality of the complex. We sought to test this hypothesis by testing pre-let-7a-1 and its CTL mutants using an in vitro uridylation assay (Figure S1). Pre-let-7a-1 mutants @2 and @3 with truncated CTLs and the naturally occurring 3′ adjacent AU dinucleotide or a UA mutation, respectively, demonstrated robust Lin28a-binding efficiency (Figures 1 and S2). Importantly, despite preserving the secondary structure and the distance of the GGAG motif from the pre-miRNA stem, as assayed by RNA structure probing with lead ions and ribonuclease T1 (Figure S3), pre-let-7a-1@2 was efficiently uridylated, but pre-let-7a-1@3 was not (Figure 1C). Pre-let-7a-1 mutant @1 with a minimal terminal loop harboring the wild-type GGAGAU sequence did not bind Lin28a (Figure 1B), nor was it uridylated (Figure 1C). These data point to structural RNA constraints on Lin28a’s interaction with its targets.

**Figure 2:**
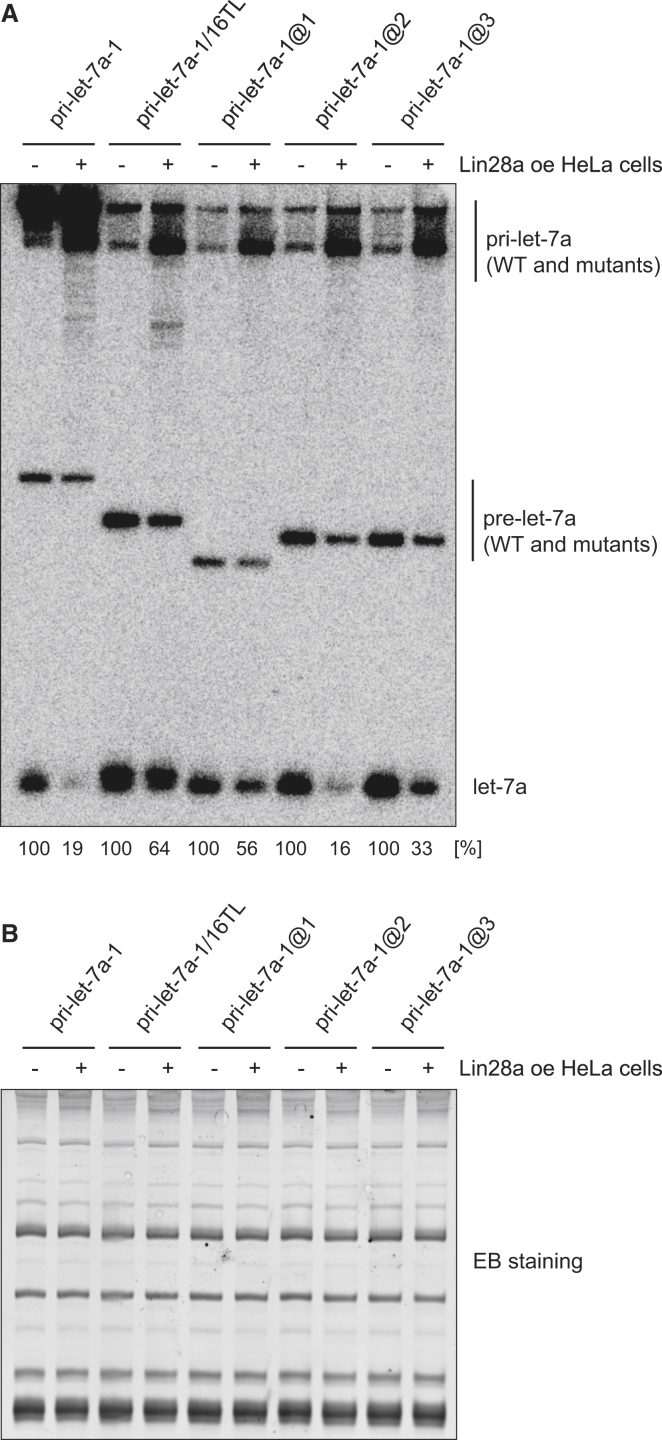
Lin28a Inhibits Pri- and Pre-let-7 Processing, but the Efficiency Depends on Fine Features of the Let-7 CTL (A) Northern blot analysis with a probe for let-7a of total RNA from HeLa cells transfected with only a pCG plasmid coding for pri-let-7a-1 or one of its CTL mutants or cotransfected with a pri-let-7 plasmid and pCG T7-Lin28a. (B) As a loading control, ethidium bromide (EB) staining of the polyacrylamide gel before blotting is shown.

Co-overexpression of pri-let-7a-1 mutants with Lin28a in HeLa cells followed by northern blotting against let-7a confirmed the importance of the AU dinucleotide adjacent to the GGAG motif in the Lin28a-mediated inhibition of let-7a processing, as only the pri-let-7a-1@2 mutant was suppressed to the same extent as the wild-type pri-let-7a-1 (Figure 2). Notably, all substrates were inhibited at the Drosha cleavage step (Figure 2), validating previous claims regarding Lin28a-mediated repression of pri-let-7 processing (Viswanathan et al., 2008). Finally, introduction of an AU dinucleotide adjacent to the GGAG motif in the terminal loop of pre-miRNA-363, which previously had been shown to bind Lin28a but undergo only marginal uridylation (Heo et al., 2009), greatly enhanced its uridylation without changing its affinity for Lin28a (Figures 1D–1F). Notably, the uridylation efficiency of pre-miRNA-363@1 was much weaker than that of pre-let-7a-1 (Figures 1C and 1F). Overall, these results indicate that Lin28a binding per se, even at the same distance to the stem as in wild-type pre-let-7, is not sufficient to trigger efficient uridylation. The functional role of the AU dinucleotide adjacent to the GGAG motif suggests the existence of an additional RNA-specific cofactor for Lin28a-mediated uridylation.

### Trim25 Binds to Pre-let-7 and Interacts with Components of the Lin28/TuT4 Pathway

**Figure 3:**
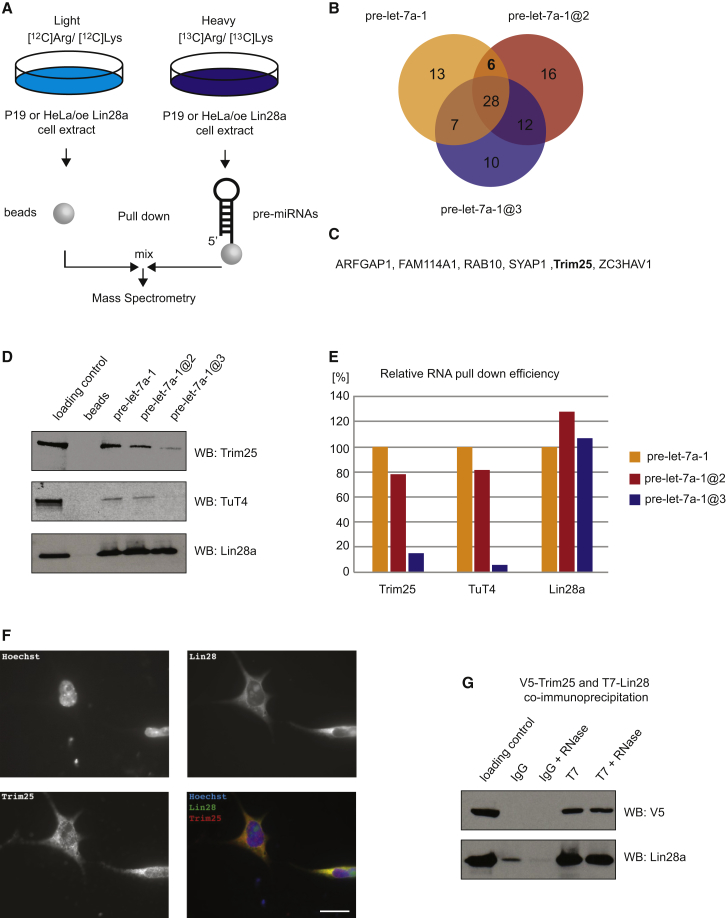
SILAC Combined with RNA Pull-Down and Mass Spectrometry Reveals Putative Cofactors for Lin28a-Mediated Uridylation (A) Schematic of the method. P19 cells or HeLa cells overexpressing Lin28a were grown in “light” medium containing 12C6-arginine and 12C6-lysine or in “heavy” medium containing 13C6-arginine and 13C6-lysine. Next, RNA pull-down was performed with either agarose beads incubated with extract from light HeLa cells or beads with wild-type pre-let-7a-1 or its CTL mutants covalently linked incubated with extract from heavy HeLa cells. After thorough washing, the resulting supernatants were mixed and subjected to quantitative mass spectrometry. (B) Venn diagrams representing numbers of overlapping proteins identified in the pull-downs with wild-type pre-let-7a-1, pre-let-7a-1@2, and pre-let-7a-1@3. (C) Proteins identified exclusively in the pre-let-7a-1 and pre-let-7a-1@2 pull-downs. (D) Western blot (WB) analysis of Trim25, TuT4, and Lin28a proteins in RNA pull-downs from extract of HeLa cells overexpressing Lin28a using wild-type pre-let-7 and its CTL mutants @2 and @3. (E) Quantification of the results presented in (D). The band intensities were calculated with ImageJ software and were normalized to 100% based on the wild-type pre-let-7a-1 pull-down. (F) Immunofluorescence staining of Hoechst (blue), Lin28a (green), and Trim25 (red) in P19 cells showing colocalization of Lin28a and Trim25 predominantly in the cytoplasm. Scale bar, 10 μm. (G) Coimmunoprecipitation between Trim25 and Lin28a was performed with cell extracts prepared from HeLa cells overexpressing T7-Lin28a and V5-Trim25. The extracts were incubated with anti-T7 or control immunoglobulin G (IgG) antibody bound to protein-A beads. The bound proteins were analyzed by western blotting with anti-Lin28a or anti-V5. To control for directionality of the interaction, the assay was also performed in the presence of RNase.

To find the RNA-specific cofactor for Lin28a-mediated uridylation, we next used RNA pull-down combined with SILAC and high-throughput mass spectrometry (Figure 3A). Pre-let-7a-1, pre-let-7a-1@2, and pre-let-7a-1@3 pulled down 28 common proteins, including Lin28a, with very similar efficiency (Figure 3B). Six proteins were pulled down specifically by wild-type pre-let-7a-1 and the @2 mutant (Figures 3B and 3C). Among them, we identified the Trim25 protein with well-documented E3 ligase function (Gack et al., 2007; Inn et al., 2011), which has recently been observed to bind RNA and be downregulated during retinoic acid-driven differentiation (Kwon et al., 2013). We validated Trim25 binding to the wild-type pre-let-7a-1 and pre-let-7a-1@2 mutant by RNA pull-down followed by western blotting (Figure 3D). The binding of Trim25 to the pre-let-7a-1@3 mutant was detectable but much weaker compared to wild-type pre-let-7a-1 and the pre-let-7a-1@2 mutant (Figures 3D and 3E). Strikingly, despite all substrates pulling down comparable amounts of Lin28a, only the wild-type and mutant pre-let-7a-1@2 successfully pulled down TuT4 (Figures 3D and 3E). TuT4 was not identified in our original RNA pull-down screen, but this could be due to lack of detection during the high-throughput mass spectrometry. Intriguingly, even though pre-miRNA-363@1 pulled down more TuT4 than wild-type pre-miRNA-363, we detected similar binding of Trim25 to both substrates (Figure S4). Importantly, relative to the loading control, pull-down of both TuT4 and Trim25 by pre-miRNA-363@1 was much less efficient than that observed for pre-let-7a-1 (Figures S4 and 3D). This suggests a different composition of the functional pre-miRNA-363 and pre-let-7-protein complexes and their putative roles in uridylation.

Next, we sought to establish whether Trim25 interacts with components of the pre-let-7/Lin28/TuT4 pathway. Using immunocytochemistry on mouse teratocarcinoma P19 cells, we found that Lin28a and Trim25 colocalize in vivo, supporting their functional link (Figure 3F). Notably, Lin28a and Trim25 localize to the cytoplasm as well as to the nucleus. Finally, using coimmunoprecipitation of ectopically expressed T7-Lin28a and V5-Trim25 in HeLa cells, we validated their interaction (Figure 3G). The stability of the complex in the presence of ribonuclease (RNase) points either to direct interaction, which is unlikely because mutant pre-let-7a-1@3 binds Lin28a, but not Trim25 (Figure 3D), or close proximity binding on the same RNA. Altogether, our results show that Trim25 could function as an RNA-specific cofactor for the Lin28a-mediated uridylation of pre-let-7.

### Trim25 Is an RNA-Specific Cofactor for Lin28a/TuT4-Mediated Uridylation

**Figure 4:**
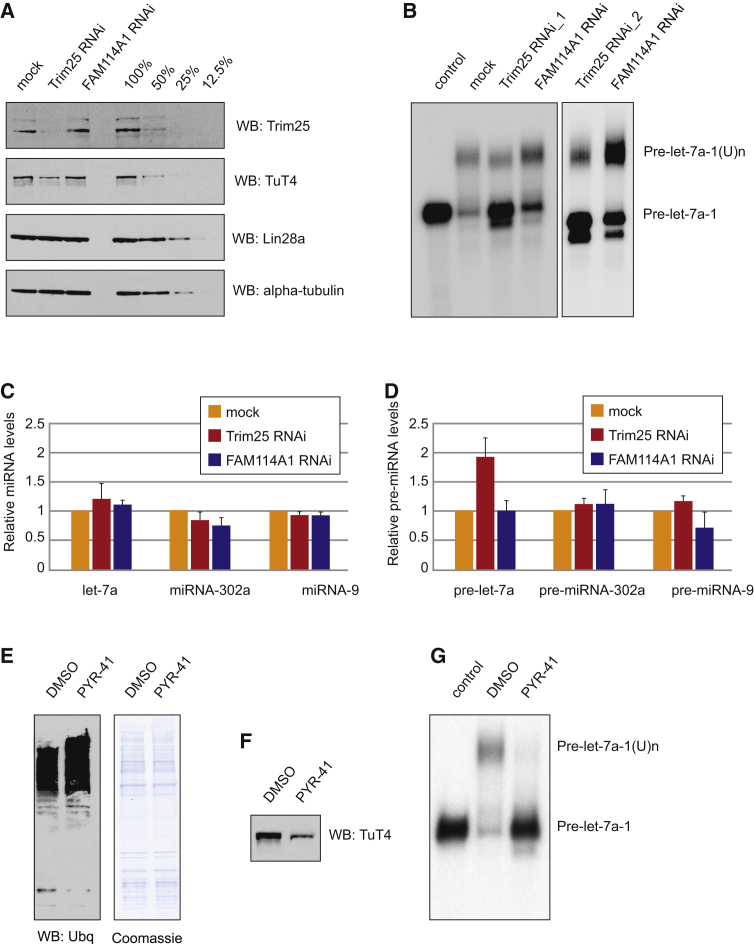
Trim25 Is a Positive Cofactor for the Lin28a-Mediated Uridylation of Pre-let-7 (A) Western blot analysis of Trim25, TuT4, Lin28a, and α-tubulin proteins in protein extracts from P19 cells depleted of Trim25 or FAM114A1 using RNAi. Serial dilutions of the total protein extracts provide estimates of the linearity and limit of detection of the western blot assay. (B) In vitro processing assays with internally radiolabeled pre-let-7a-1 transcripts (50 × 10^3^ cpm, approximately 20 pmol) in the presence of mock-, Trim25-, or FAM114A1-depleted P19 cell extract. Trim25 RNAi_1 and Trim25 RNAi_2 represent results from treatment with two different siRNA sets. The reactions were supplemented with 0.25 mM UTP. (C) Real-time quantitative RT-PCR of miRNAs (let-7a, miRNA-302a, and miRNA-9) in P19 cells depleted of Trim25 or FAM114A1. The values were normalized to miRNA-16 levels. The fold change in the abundance miRNAs mediated by RNAi was plotted relative to values from a mock-transfected control, which were set to one. Mean values and SDs of three independent experiments are shown. (D) Real-time quantitative RT-PCR of pre-miRNAs (pre-let-7a, pre-miRNA-302a, and pre-miRNA-9) in P19 cells depleted of Trim25 or FAM114A1. The values were normalized to pre-miRNA-16 levels. The fold change in the abundance of the corresponding miRNA mediated by RNAi was plotted relative to values from a mock-transfected control, which were set to one. Mean values and SDs of three independent experiments are shown. (E) Western blot analysis of ubiquitinated proteins in protein extracts from P19 cells treated with DMSO or 50 μM PYR-41 for 4 hr. As a loading control, Coomassie blue stain is shown. (F) Western blot analysis of TuT4 in total extracts from P19 cells treated with DMSO or 50 μM PYR-41 for 4 hr. (G) In vitro processing assays with internally radiolabeled pre-let-7a-1 transcripts (50 × 10^3^ cpm, approximately 20 pmol) in the presence of P19 cell extract treated with DMSO or 50 μM PYR-41 for 4 hr.

To determine whether Trim25 plays a role in Lin28a-mediated uridylation, we performed Trim25 RNAi in mouse teratocarcinoma P19 cells (Figure 4A). Efficient knockdown of Trim25 caused a noticeable decrease in TuT4 levels (Figure 4A), which suggests that Trim25 has a positive effect on the TuT4 protein level in vivo. Importantly, pre-let-7a-1 uridylation in extracts derived from cells depleted of Trim25 (by two independent small interfering RNA [siRNA] substrates) was inhibited as seen by substantial retention of unprocessed pre-let-7a substrate (Figure 4B). Furthermore, Trim25 knockdown did not affect mature miRNA levels but caused 2-fold accumulation of pre-let-7a (Figures 4C and 4D). This mirrors the effects reported for TuT4 knockdown (Chang et al., 2013). Crucially, neither pre-miRNA-302a nor pre-miRNA-9, which were previously shown to bind Lin28a, but not undergo uridylation (Nowak et al., 2014; Wilbert et al., 2012), were affected by Trim25 knockdown (Figures 4C and 4D). This provides evidence that Trim25 is an RNA-specific cofactor stimulating Lin28a-mediated uridylation.

### Inhibition of the Proteasome and Ubiquitin-Activating Enzyme E1 Results in Decreased Levels and Activity of TuT4

Because Trim25 is an E3 ubiquitin ligase, we sought to determine whether interfering with the ubiquitination pathway would inhibit its downstream activity on the Lin28a/TuT4 pathway. After treating P19 cells with the E1 ubiquitin-activating enzyme inhibitor PYR-41 (Yang et al., 2007) for 4 hr, we detected a significant reduction in TuT4 levels, albeit with similar levels of total protein (Figures 4E and 4F). Crucially, extracts derived from PYR-41-treated cells were impaired in Lin28-mediated uridylation of pre-let-7a-1 (Figure 4G). This indicates that a functional ubiquitination pathway is necessary for TuT4 stability. Altogether, these results imply the existence of a connection between canonical RNA binding proteins involved in RNA processing and RNA binding factors involved in protein modification.

## Discussion

RNA binding proteins control gene expression programs in living cells by regulating RNA processing, modification, localization, translation, and turnover (Glisovic et al., 2008). They predominantly recognize RNA sequences several nucleotides long that occur frequently in various transcriptomes (Ray et al., 2013). For example, SRSF1 and SRSF2 were found to bind ∼50,000 specific sequences in mammalian cells (Pandit et al., 2013). Furthermore, large numbers of RNA binding proteins are multifunctional, and even more strikingly, depending on the substrate they bind, they have opposite effects on the same RNA-processing event. It has been previously shown that the multifunctional protein hnRNP A1 stimulates pri-miRNA-18a processing (Guil and Cáceres, 2007; Michlewski et al., 2008, 2010) but inhibits the biogenesis of pri-let-7a (Michlewski and Cáceres, 2010a). Furthermore, Drosha has been recently shown to promote alternative splicing of exon 5 of eIF4H gene in cleavage-independent manner (Havens et al., 2014). Finally, many high-affinity RNA-protein interactions are functionally passive (Singh and Valcárcel, 2005). This raises the important question of what determines the functionality of RNP complexes.

The RNA binding protein Lin28a is a pluripotency-promoting factor involved in downregulation of let-7 during early development and differentiation in metazoans (Nowak and Michlewski, 2013; Shyh-Chang and Daley, 2013). The major mechanism involves binding to the CTL of pre-let-7 via the cold shock domain (CSD) and CHCC zinc-finger domain (Choudhury and Michlewski, 2012; Loughlin et al., 2012; Nam et al., 2011) and subsequent binding of TuT4 or the closely related TuT7, which adds an oligo-U tail to the 3′ end of pre-let-7 (Hagan et al., 2009; Heo et al., 2009; Thornton et al., 2012). Overexpression of Lin28a is linked with induction of tumor growth (Urbach et al., 2014). Lin28 preferentially interacts with GGAG and GNGAY motifs (Wilbert et al., 2012), and it binds to uridine-rich elements and isolated guanosines (Hafner et al., 2013). Importantly, a number of pre-miRNAs, such as pre-miRNA-363, pre-miRNA-302d, and pre-miRNA-9-1, contain the highly conserved GGAG motif and bind Lin28a with high affinity but are not uridylated (Heo et al., 2009; Nowak et al., 2014; Wilbert et al., 2012). This strongly suggests the existence of substrate-specific coactivators or inhibitors of Lin28a function.

Using RNA pull-down coupled with quantitative SILAC mass spectrometry (Choudhury et al., 2013; Michlewski and Cáceres, 2010b), we identified the E3 ligase Trim25 as an RNA-specific cofactor for the Lin28a/TuT4 uridylation pathway. Trim25 is a member of the tripartite motif (TRIM) protein family, which is involved in the innate immune response (Versteeg et al., 2013). Its main protein target is retinoic acid-inducible gene I (RIG-I), the ubiquitination of which leads to type I interferon production (Inn et al., 2011). Recently, Trim25 has been shown to bind RNA through its coiled-coil (CC) domain and be downregulated during retinoic acid-driven differentiation (Kwon et al., 2013). Interestingly, another protein from the same family, Trim71, has previously been implicated in the miRNA pathway due to its targeting of Lin28b and Ago2 for canonical proteasome-dependent degradation (Lee et al., 2014; Rybak et al., 2009). Here we show that an E3 ligase can recognize specific RNA and positively regulate a protein involved in the processing of cognate RNA in *cis*. Our model is that the sequence and structural features of the pre-let-7 CTL allow efficient Trim25 binding, which in turn stabilizes TuT4 leading to increased uridylation. In the future, it will be important to determine which type of modifications are responsible for this phenomenon and whether other members from the Trim family have similar RNA-specific functions. Because there are 75 Trim proteins annotated in the human genome, functional overlap, redundancy, and specification are very likely (Versteeg et al., 2013). Furthermore, pre-miRNAs, which possess efficient Lin28a binding but are not uridylated, could lack Trim25 binding, as seen in the case of pre-let-7a-1@3. Finally, the existence of additional factors inhibiting Lin28a function on non-pre-let-7 substrates cannot be ruled out.

In summary, we have identified a process with extensive implications for all RNA-protein interactions. Protein-modifying enzymes such as kinases, phosphatases, and E3 ligases with the ability to bind RNA can provide selectivity for RNA processing events, spatially modulating the activity of canonical RNP complexes.

## Experimental Procedures

### Cell Culture

Mouse teratocarcinoma P19 cells or HeLa cells (American Type Culture Collection) were maintained in standard Dulbecco’s modified Eagle’s medium (DMEM) (Life Technologies), supplemented with 10% fetal bovine serum (Life Technologies). For SILAC mass spectrometry, undifferentiated cells were cultured in DMEM supplemented with “heavy” [13C]Arg/[13C]Lys isotopes, and differentiation was performed using DMEM supplemented with “light” [12C]Arg/[12Lys] isotopes (Pierce SILAC Proteins Quantitation Kit, Thermo Scientific). Where indicated, cells were treated with the E1 ubiquitin-activating enzyme inhibitor PYR-41 (Yang et al., 2007) for 4 hr.

### In Vitro Transcription and Pre-miRNA Uridylation Assays

Pre-miRNA transcripts were prepared by standard in vitro transcription with T7 RNA polymerase in the presence of [α-32P]-uridine-5'-triphosphate (UTP). Where indicated, pre-miRNA probes were 5′ end-labeled with [γ-32P]-ATP. The templates used to generate the transcripts were prepared by PCR amplification from larger fragments of the human genome using primers corresponding precisely to the ends of pre-miRNAs. The pre-let-7a-1@1, pre-let-7a-1@2, pre-let-7a-1@3, and pre-miRNA-363@1 templates were derived using mutagenesis of the wild-type pre-miRNAs. Generation of the template for pre-let-7a-1@16TL was described before (Nowak et al., 2014). Gel-purified probes (50 × 10^3^ counts per minute [cpm]), approximately 20 pmol) were incubated in 30 μl reaction mixtures containing 50% (v/v) total P19 cell extract (approximately 10 μg × μl^−1^), 0.5 mM ATP, 20 mM creatine phosphate, and 3.2 mM MgCl_2_. The reactions were supplemented with 0.25 mM UTP and incubated at 37°C for 30 min followed by phenol-chloroform extraction, precipitation, and 8% (w/v) denaturing gel electrophoresis. The bands on the gel were visualized with a radiographic film or by exposure to a phosphorimaging screen and subsequent scanning on a FLA-5100 scanner (Fujifilm).

### RNA Pull-Down and Mass Spectrometry

RNA pull-down and mass spectrometry were performed as previously described (Choudhury et al., 2013), with slight modifications. In brief, total protein extracts from undifferentiated or differentiated P19 cells grown in light [12C]Arg/[12Lys] and heavy [13C]Arg/[13C]Lys isotopes, respectively, were incubated with in vitro-transcribed RNAs chemically coupled to agarose beads. The incubation was followed by a series of washes with buffer G (20 mM Tris [pH 7.5], 135 mM NaCl, 1.5 mM MgCl_2_, 10% (v/v) glycerol, 1 mM EDTA, 1 mM dithiothreitol, and 0.2 mM phenylmethanesulfonylfluoride). After the final wash, the proteins associated with the beads were analyzed by SDS-PAGE followed by in-gel digestion and mass spectrometry or western blotting.

### Western Blot Analysis

Total protein samples (100 μg per lane), isolated by sonication, were resolved by standard NuPAGE SDS-PAGE electrophoresis with MOPS running buffer (Life Technologies) and transferred onto a nitrocellulose membrane. The membrane was blocked overnight at 4°C with 1:10 western blocking reagent (Roche) in Tris-buffered saline buffer with 0.1% Tween-20 (TBST). The following day, the membrane was incubated for 1 hr at room temperature with primary antibody solution in 1:20 western blocking reagent diluted in TBST: rabbit polyclonal anti-Lin28a (A177) (1:1,000, Cell Signaling Technology), rabbit polyclonal anti-Trim25 (1:1,000, Abcam), rabbit polyclonal anti-TuT4 (1:1,000, Protein-Tech), rabbit polyclonal anti-DHX9 (1:1,000, Protein-Tech), mouse-monoclonal anti-ubiquitin (1:1,000 Cell Signaling Technology), and mouse-monoclonal anti-β-tubulin (1:10,000, Sigma-Aldrich). After washing in TBST, the blots were incubated with the appropriate secondary antibody conjugated to horseradish peroxidase and detected with SuperSignal West Pico detection reagent (Thermo Scientific). The membranes were stripped using ReBlot Plus Strong Antibody Stripping Solution (Chemicon) equilibrated in water, blocked in 1:10 western blocking solution in TBST, and reprobed as described above.

### Northern Blot Analysis

Northern blot analysis was performed as previously described (Choudhury et al., 2013). In brief, total RNA was resolved on a 10% PAGE-urea gel. The ribosomal RNA was visualized with ethidium bromide to confirm equal loading. The RNA was transferred onto a nitrocellulose membrane (Hybond N). The membrane was prehybridized overnight at 40°C with 10 ml of hybridization buffer (1 × saline sodium citrate [SSC], 1% SDS, 200 μg × ml^−1^, single-stranded DNA). A northern probe was prepared using the mirVana miR Probe Construction Kit (Life Technologies) and hybridized against the membrane for 2 hr at 40°C in 10 ml of hybridization buffer. Subsequently, the membrane was washed with wash buffer (0.2% SSC, 0.2% SDS). The signal was registered with radiographic film or exposed to a phosphorimaging screen and scanned on a FLA-5100 scanner (Fujifilm).

### RNAi, miRNA, and Protein Overexpression

Pools of siRNAs were obtained from Dharmacon in the format of three independent siRNAs targeting different regions of the mRNA coding for the protein of interest. Genomic fragments containing miRNAs were cloned into the pCG T7 plasmid and transiently expressed in HeLa cells. Four micrograms of siRNA was delivered in two transfection events separated by 48 hr using nucleofection technology (AMAXA), according to the manufacturer’s instructions. HeLa cells transfections were performed using Lipofectamine 2000 (Life Technologies) according to the manufacturer’s instructions.

## Author Contributions

N.R.C. designed, performed, and analyzed the experiments and contributed to the writing of the manuscript. J.S.N. performed and analyzed the experiments. J.Z. and J.R. performed the mass spectrometry analysis. G.M. designed, performed, and analyzed the experiments, wrote the manuscript, and supervised the whole project.

## Supplemental Information

Document S1. Supplemental Experimental Procedures and Figures S1–S4

Document S2. Article plus Supplemental Information
