# Supplementary material for: Trim25 Is an RNA-Specific Activator of Lin28a/TuT4-Mediated Uridylation
Source: Cell Rep. 2014 Nov 6;9(4):1265–72. doi: 10.1016/j.celrep.2014.10.017 (PMC4542301; doi:10.1016/j.celrep.2014.10.017)
Supplement: Document S1. Supplemental Experimental Procedures and Figures S1–S4 [file mmc1.pdf]

Cell Reports, Volume *9*

Supplemental Information

Trim25 Is an RNA-Specific Activator of Lin28a/TuT4-Mediated Uridylation

Nila Roy Choudhury, Jakub S. Nowak, Juan Zuo, Juri Rappsilber, Steven H. Spoel, and Gracjan Michlewski

SUPPLEMENTAL INFORMATION

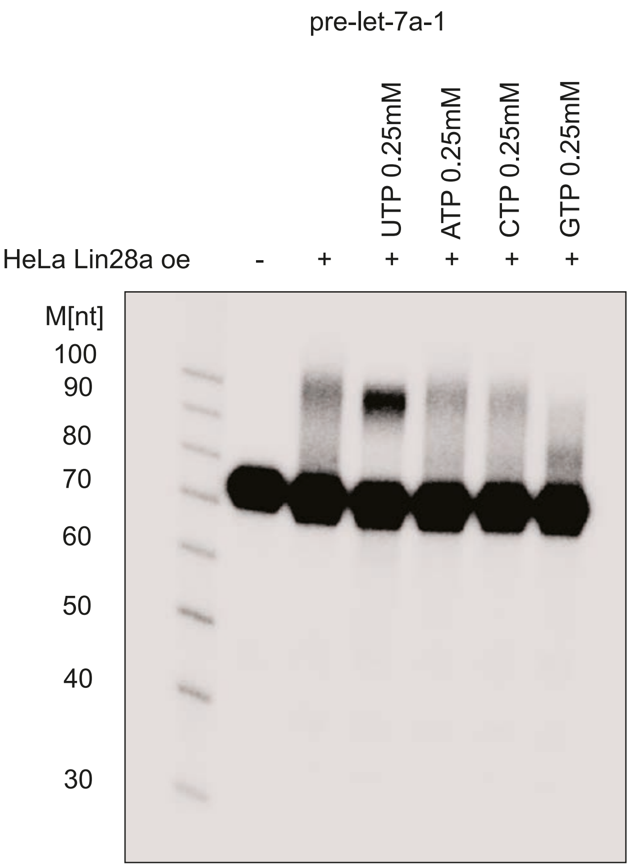

Supplemental Figure 1. Related to Figure 1. Pre-let-7a-1 is efficiently uridylated in extract from HeLa cells overexpressing Lin28a. In vitro processing uridylation assays performed with internally radiolabeled pre-let-7a transcripts (50×10^3 c.p.m., approximately 20 pmol) in the presence of extract derived from HeLa cells overexpressing Lin28a. (−) represents an untreated control. The reactions were supplemented with 0.25 mM of either UTP, ATP, CTP or GTP.

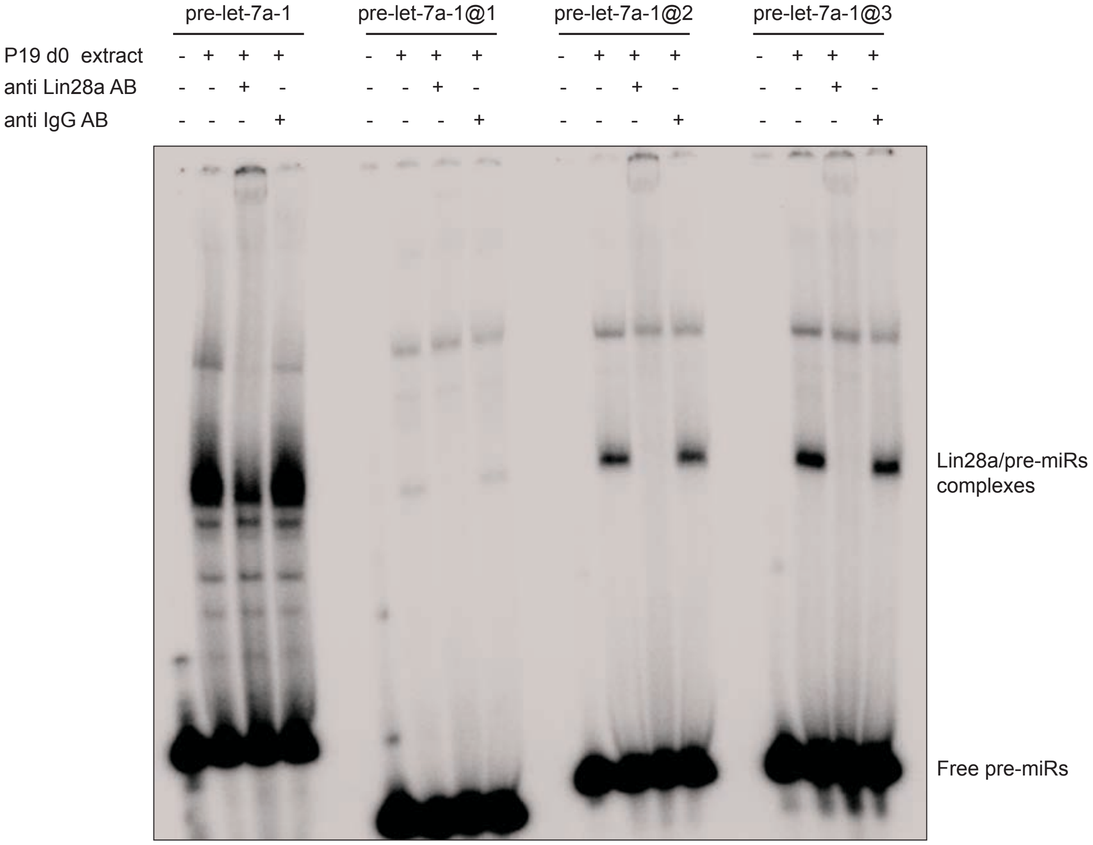

Supplemental Figure 2. Related to Figure 1. Lin28a efficiently binds pre-let-7a-1, pre-let-7a-1@2 and pre-let-7a-1@3 but not pre-let-7a-1@1. EMSA analysis of pre-let-7a-1 and CTL mutants in extracts from d0 P19 cells. Where indicated, either anti-Lin28a or IgG antibody was added to the reaction mixture at 1:100. Lin28a/pre-miRNA complexes are indicated by bands, the intensity of which is decreased upon addition of anti-Lin28a antibody.

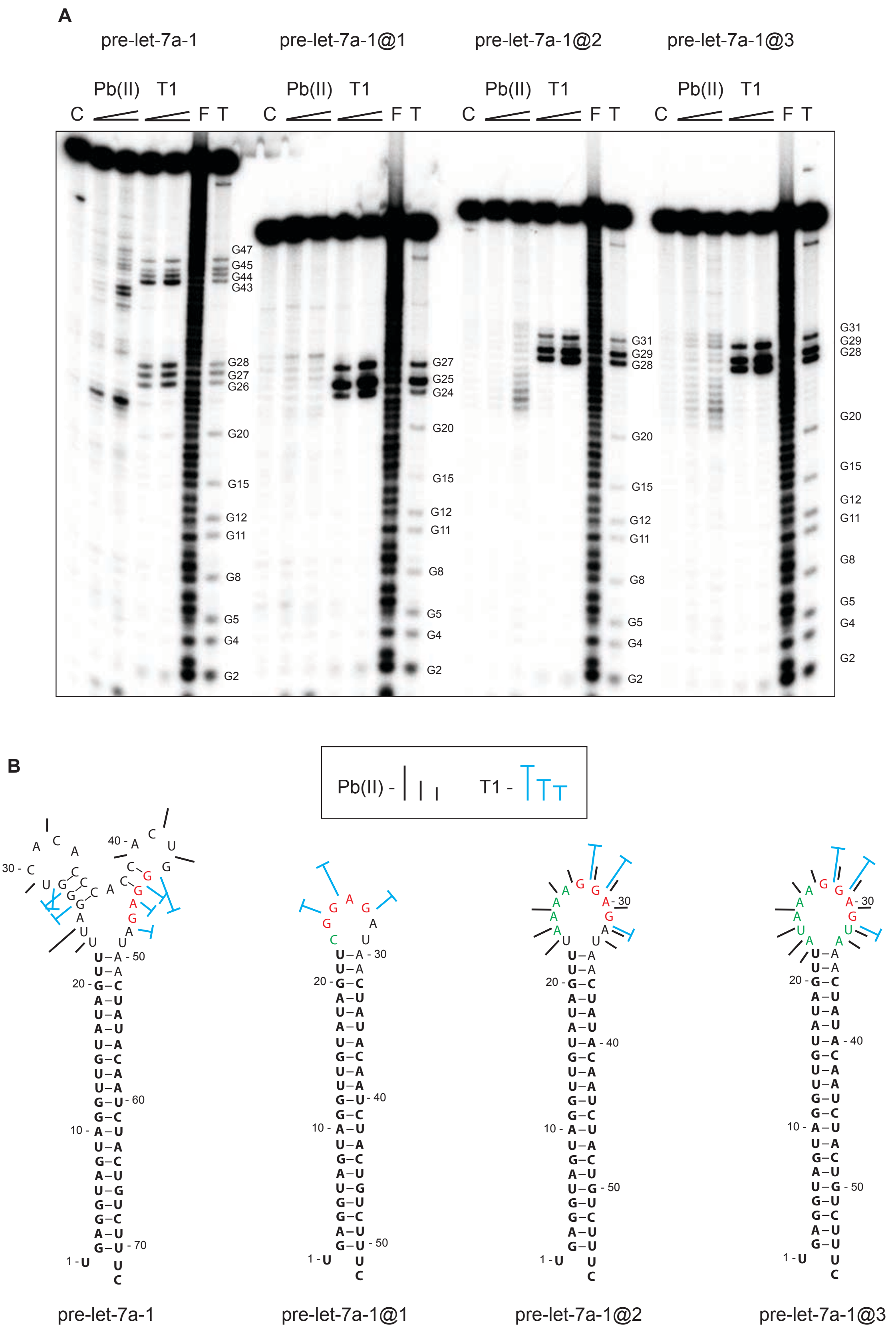

A
pre-let-7a-1
pre-let-7a-1@1
pre-let-7a-1@2
pre-let-7a-1@3
Pb(II)
T1
C
F
T
G47
G45
G44
G43
G28
G27
G26
G25
G24
G31
G29
G20
G15
G12
G11
G8
G5
G4
G2
B
Pb(II) -
T1 -
pre-let-7a-1
pre-let-7a-1@1
pre-let-7a-1@2
pre-let-7a-1@3

Supplemental Figure 3. Related to Figure 1. RNA structure probing reveals that pre-let-7a-1@2 and pre-let-7a-1@3 are structurally identical. The results of structure probing of pri-let-7a-1, pre-let-7a-1@1, pre-let-7a-1@2 and pre-let-7a-1@3 are shown. Cleavage patterns were obtained for 5′ 32P-labeled pri-let-7 transcripts (100×10^3 cpm, approximately 40 pmol) treated with Pb(II) ions (0.5, 1 mM) or ribonuclease T1 (1.5, 3.0 U/μL). Lanes F and T denote nucleotide residues subjected to partial digestion with formamide (every nucleotide) or ribonuclease T1 (G-specific cleavage), respectively. Electrophoresis was performed on an 8% polyacrylamide gel under denaturing conditions. The positions of selected G residues are indicated. Nucleotides are numbered from the 5′ end of the Drosha cleavage site. (B) Proposed structures of pri-let-7a-1, pre-let-7a-1@1, pre-let-7a-1@2 and pre-let-7a-1@3. The cleavage sites generated by the structure probes and their intensities are shown.

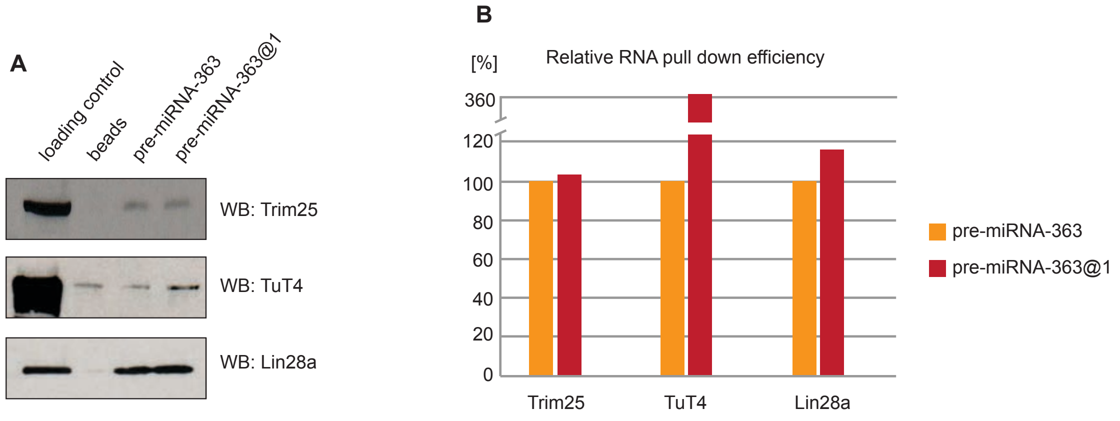

Supplemental Figure 4. Related to Figure 3. Pre-miRNA-363 binds Trim25 but notably less efficiently than pre-let-7a-1 (Figure 3D, E). (A) Western blot analysis of Trim25, TuT4 and Lin28a proteins in RNA pull-downs from extract of HeLa cells overexpressing Lin28a using wild-type pre-miRNA-363 and the pre-miRNA-363@1 CTL mutant. (E) Quantification of the results presented in A. The band intensities were calculated with ImageJ software and were normalized to 100% based on the wild-type pre-miRNA-363 pull-down.

SUPPLEMENTAL EXPERIMENTAL PROCEDURES

Immunofluorescence

Trim25 was visualized in P19 cells using primary monoclonal rabbit anti-human Trim25 antibody [EPR7315] from Abcam (ab167154). Lin28a was detected by native fluorescence of GFP molecules present in the Lin28GFP transgene. Prior to microscopy, cells were plated on cover slips coated with 2 mL of 10 μg/mL PDL (Sigma-Aldrich P4707). At 24 h after plating, the cells were washed with PBS and fixed with 4% formaldehyde (Sigma-Aldrich 37% w/v in H2O 252549-500) for 10 minutes at RT. Next, the cells were permeabilized for 10 min at RT by adding 0.2% Triton-X (Sigma-Aldrich T9284-100). Subsequently, the cells were blocked for 15 min at RT with goat serum and incubated for 1 h at RT with primary antibody at a 1/1000 dilution in goat serum and for 1 h at RT with Alexa Fluor goat anti-rabbit 568 secondary antibody (Molecular Probes A-11036) at a dilution of 1/1000 in goat serum. In the last step, cells were counterstained with Hoechst dye (1/20000) for 15 min at RT and mounted on slides using 15 μL of mounting medium (Molecular Probes Prolong Gold AntiFade P36930). Each of the above steps was separated by 3 washes with PBS for 5 min at RT. Mounted cells were visualized using a Zeiss Axio Imager Z1 fluorescent microscope with an apotome and analyzed with ImageJ software.

Co-immunoprecipitation

Extracts prepared from HeLa cells transfected with pCG T7-Lin28a and V5-Trim25 were incubated overnight in the cold room with anti-T7 antibody or control IgG antibody bound to protein-A beads and washed three times with buffer D. The bound proteins were separated on a 10% SDS–polyacrylamide gel and analyzed by western

blotting with anti-Lin28a or anti-V5 antibodies. Alternatively, the immunoprecipitate was treated with RNases A/T1 for 30 min prior to loading on the gel. The loading control represents 2% of the amount of extract used for each immunoprecipitation.

Real-time Quantitative RT-PCR

Real-time quantitative reverse transcription (qRT)–PCR was performed using the SuperScript III Platinum SYBR Green One-Step qRT–PCR Kit (Life Technologies) following the manufacturer's instructions on a Roche 480 LightCycler. For mature miRNAs we used miScript qRT–PCR kit (Qiagen) on total RNA isolated with TRIzol reagent (Life Technologies), and each sample was run in duplicate. To assess the levels of the corresponding miRNAs, their values were normalized to that of miRNA-16. For each measurement, three independent experiments were performed. For miRNA precursors we used the miScript qRT–PCR kit (Qiagen) on RNA size-fractionated through 6% PAGE–urea gel purification and each sample was run in duplicate. To assess the levels of the corresponding miRNAs, their values were normalized to that of pre-miRNA-16. For each measurement, three independent experiments were performed.

Electrophoretic Mobility Shift Assays

Electrophoretic mobility shift assays (EMSAs) were performed as previously described (Nowak et al., 2014). Internally labeled pre-miRNAs were incubated in a 30 μl reaction mixture containing 50% (v/v) total P19 cell extract (approximately 10 μg μl^{-1}), 0.5 mM ATP, 20 mM creatine phosphate and 3.2 mM MgCl2. The reactions were incubated at 4°C for 1 h followed by electrophoresis in a 6% (w/v) non-denaturing gel. Where indicated, antibodies were added to reaction mixture

(1:100) to generate a supershift. The signal was registered with radiographic film or exposed to a phosphorimaging screen and scanned on a FLA-5100 scanner (Fujifilm).

RNA Structure Probing

RNA structure probing was performed as previously described (Choudhury et al., 2013). Briefly, pre-miRNA transcripts were 5' end-labeled with T4 polynucleotide kinase (NEB) and [gamma-32P] ATP (4,500 Ci/mmol; Amersham Pharmacia Biotech). RNAs were denaturated in a buffer containing 1.2 mM MgCl2, 48 mM NaCl, 12 mM Tris–HCl pH 7.2 and then renaturated by slowly cooling to 37°C. Limited RNA digestion was initiated by mixing 8 μl of the RNA sample (100×10^3 c.p.m., approximately 40 pmol, 4 μM) with 2 μl of a probe solution (Pb(II) ions or ribonuclease T1). The reactions were performed at 37°C for 10 min. To determine the cleavage sites, the products of alkaline hydrolysis and limited ribonuclease T1 digestion of the 5' end-labeled RNA fragmentation reaction were separated on a 10% PAGE–urea gel along with the structure-probing products. Electrophoresis was performed at 1500 V and was followed by autoradiography at -80°C with an intensifying screen or exposure to a PhosphorImager screen (Molecular Dynamics).

REFERENCES

Choudhury, N.R., de Lima Alves, F., de Andres-Aguayo, L., Graf, T., Caceres, J.F., Rappsilber, J., and Michlewski, G. (2013). Tissue-specific control of brain-enriched miR-7 biogenesis. Genes & development *27*, 24-38.

Nowak, J.S., Choudhury, N.R., de Lima Alves, F., Rappsilber, J., and Michlewski, G. (2014). Lin28a regulates neuronal differentiation and controls miR-9 production. Nature communications *5*, 3687.
